# Developmental Regulation and Spatiotemporal Redistribution of the Sumoylation Machinery in the Rat Central Nervous System

**DOI:** 10.1371/journal.pone.0033757

**Published:** 2012-03-16

**Authors:** Céline Loriol, Joséphine Parisot, Gwénola Poupon, Carole Gwizdek, Stéphane Martin

**Affiliations:** 1 Institut de Pharmacologie Moléculaire et Cellulaire, Centre National de la Recherche Scientifique, Valbonne, France; 2 University of Nice - Sophia-Antipolis, Nice, France; University of Toronto, Canada

## Abstract

**Background:**

Small Ubiquitin-like MOdifier protein (SUMO) is a key regulator of nuclear functions but little is known regarding the role of the post-translational modification sumoylation outside of the nucleus, particularly in the Central Nervous System (CNS).

**Methodology/Principal Findings:**

Here, we report that the expression levels of SUMO-modified substrates as well as the components of the sumoylation machinery are temporally and spatially regulated in the developing rat brain. Interestingly, while the overall sumoylation is decreasing during brain development, there are progressively more SUMO substrates localized at synapses. This increase is correlated with a differential redistribution of the sumoylation machinery into dendritic spines during neuronal maturation.

**Conclusions/Significance:**

Overall, our data clearly demonstrate that the sumoylation process is developmentally regulated in the brain with high levels of nuclear sumoylation early in the development suggesting a role for this post-translational modification during the synaptogenesis period and a redistribution of the SUMO system towards dendritic spines at a later developmental stage to modulate synaptic protein function.

## Introduction

Neurons are highly specialized cells whose connectivity at synapses enables rapid information transfer in the brain. Synapse formation and elimination as well as synaptic transmission and plasticity largely depend on the correct targeting and arrangement of complex protein networks on both sides of the synapse. These networks are organized in an array of scaffolding and adaptors molecules, presenting multiple protein-protein interaction domains to anchor and position effectors such as neurotransmitter receptors or components of signaling pathways and their associated regulators. The structure and composition of synaptic networks and effectors activities are highly regulated during developmental processes and are also dynamically modified to modulate synaptic transmission and plasticity. Recent developments in proteomics have provided a global identification of proteins organizing these synaptic networks. However, the spatiotemporal and functional regulation of these protein complexes is still largely unknown. These dynamic processes are often regulated by post-translational modifications (PTM) such as phosphorylation or ubiquitination [1]. Interestingly, sumoylation is now emerging as a potent post-translational mechanism to regulate synaptic formation and plasticity.

Sumoylation was identified fifteen years ago [2] and consists in the covalent labelling of the Small Ubiquitin-like MOdifier SUMO (100 amino acid protein, ∼11 kDa) to specific lysine residues of target proteins. Four mammalian SUMO paralogs (SUMO1-4) have been identified so far. SUMO1-3 are ubiquitously expressed whereas SUMO4 is poorly characterized and mainly expressed in kidney and spleen, [3], [4], [5]. SUMO2 and SUMO3 are almost identical and referred as SUMO2/3. SUMO1 shares only 47% identity with SUMO2/3 and unlike SUMO2/3 cannot form poly-SUMO chains [6]#.

The covalent attachment of SUMO to target proteins is mediated through an enzymatic cascade. SUMO precursors are first matured by the hydrolase activity of desumoylation enzymes called SENPs. Matured SUMOs are then activated for conjugation in an ATP-dependent manner by the specific SUMO E1-activating complex formed by SAE1/SAE2 (also named AoS1/Uba2). SUMO is transferred onto Ubc9, the **unique** E2-conjugating enzyme of the system. Then, Ubc9 either directly or in conjunction with one of the SUMO E3 ligating enzymes catalyzes SUMO conjugation to specific lysine residues of target proteins [3], [5], [7], [8]. Despite covalent, sumoylation is readily reversible due to the isopeptidase activity of the SENP enzymes [9], [10]. In humans, six SENPs (SENP1-3 and SENP5-7) have been identified and exhibiting specific subcellular distribution and distinct specificity towards SUMO paralogs [9], [10].

Molecular consequences of sumoylation are multiple. Sumoylation may mask protein-protein interaction site, create new binding interface or lead to conformational changes. Another interesting emerging role for sumoylation in the CNS is the propensity to regulate protein aggregation [11], [12], [13].

In neurons, SUMO modification influences various aspects of neuronal activity [7], [14], [15]. Sumoylation was originally thought to target nuclear proteins but it has become clear that it also has important extranuclear roles and regulates the function of many proteins including those involved in neurological disorders. Sumoylation has also been shown to modify the stability and activity of many transcription factors to regulate neuronal morphogenesis and post-synaptic differentiation [16], [17], [18]. We reported the presence of multiple unidentified sumoylation substrates at synapses [19]#. This finding raises the intriguing possibility that sumoylation may play important roles in brain function. Since then, several cytosolic and plasma membrane proteins important for neuronal excitability and synaptic transmission were shown to be sumoylated, thereby modulating their stability, subcellular targeting, transport or interacting properties [20], [21], [22].

Although sumoylation regulates various key cellular processes, the regulatory mechanisms of the SUMO system during brain development are still largely unknown. Therefore, investigating the temporal and spatial regulation of the SUMO system in the developing brain is of particular interest to start unravelling the functional roles of sumoylation in organizing neuronal networks. Here, using brain fractionation experiments at various developmental stages, we demonstrate that there is a developmental regulation of both SUMO substrates and sumo-/desumoylation enzyme expression levels. Moreover, immunocytochemical experiments on primary cultures of rat hippocampal neurons reveal that this developmental regulation is associated with a synaptic redistribution of the sumoylation machinery during neuronal maturation. Altogether, our data indicate that the sumoylation process is highly regulated in the developing rat brain and very active during period of synaptic formation and/or stabilization.

## Results

### Developmental regulation of SUMO substrates and enzymes in the rat brain

**Figure 1:**
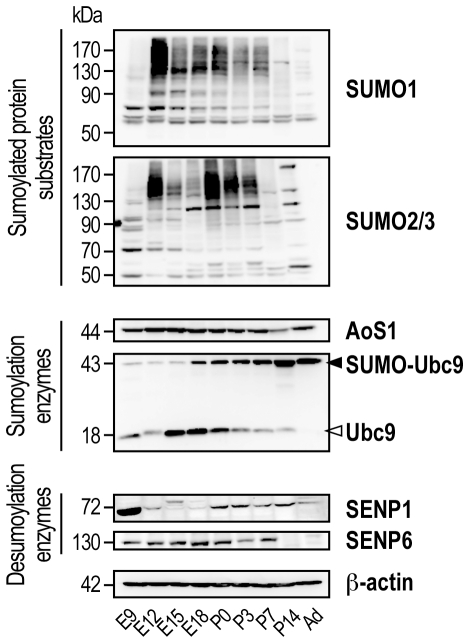
Developmental regulation of the sumoylation pathway in the rat brain. Representative developmental expression profiles of SUMO1- and SUMO2/3-modified protein substrates and sumoylation (AoS1, Ubc9) and desumoylation (SENP1 and SENP6) enzymes. Whole rat brain homogenates at different ages, ranging from the embryonic day E9 to the post-natal day P14 and the adult (Ad) stage, were prepared in the presence of NEM to protect proteins from desumoylation as described in the Method section. Lower panel shows immunoblot of standard ß-actin loading control.

The expression profiles of SUMO-modified substrates and some of the existing SUMO enzymes have been investigated in a number of cell lines *e.g.* SHSY5Y neuroblastoma [23]#, mouse [24] and drosophila germ line cells [25]. However, the developmental expression profile of protein sumoylation and the spatiotemporal regulation of the components of the sumoylation machinery in the brain have not been reported so far. We therefore examine the expression levels of SUMO-modified substrates and key sumoylation and desumoylation enzymes on proteins extracted from whole rat brain at a series of age points between the embryonic day E9 and the adult stage (**Fig. 1**).

Multiple SUMO-conjugated proteins were detected with distinct SUMO1- and SUMO2/3-conjugated protein profiles at all time point investigated (**Fig. 1**). Indeed, SUMO1-sumoylated protein substrate immunoreactivity was detected early in the development, with a sharp increase at E12 followed by a slow decline to reach a relatively low level in the adult brain. SUMO2/3-modified proteins were also developmentally regulated with a two-phase expression profile peaking respectively at E12 and birth.

Covalent SUMO modification requires the free matured SUMO to be activated prior to its conjugation to target proteins. Specific enzymes are necessary to perform these successive enzymatic steps *i.e.* the activating sumoylation complex AoS1/Uba2 and the unique SUMO-conjugating enzyme Ubc9. We show that the overall level of AoS1 remained almost unchanged throughout the brain development period (**Fig. 1**). On the contrary, the conjugation enzyme Ubc9 was developmentally regulated and appears in two forms, a ∼18 kDa unmodified free Ubc9 and a ∼40 kDa mono-sumoylated form of the enzyme on its N-terminal lysine residue K^14^ (**Fig. S1** and [26], [27]. Our data clearly show a developmental switch from the non-sumoylated Ubc9 early in the development to a SUMO-modified form of the enzyme at a later maturation stage (**Fig. 1**). Interestingly, it was reported that Ubc9 sumoylation could regulate SUMO target discrimination [26]. Thus, this switching from the non-sumoylated Ubc9 to a SUMO-modified form of the enzyme suggests that this regulatory step also occurs in the developing rat brain to finely modulate the specificity of protein sumoylation.

Despite being covalent, sumoylation is a reversible modification through the action of specific desumoylation enzymes called SENPs [10]#. SENP1 and SENP6 were chosen in this study because they are the only two enzymes expressed throughout the cells and not only in the nucleus or mitochondria as shown for other members of the SENP family [10]. Both SENP1 and SENP6 enzymes were highly expressed early in the development and were then decreased towards the adult stage (**Fig. 1**).

Together, these results suggest a role of the sumoylation process during brain development. This control of the overall protein sumoylation profile is occurring at ∼E12 which corresponds to the beginning of the synaptogenesis period in the rat brain [28]. Furthermore, the concomitant developmental regulation of sumoylation and desumoylation enzyme expression indicates that sumoylation is a dynamic regulated process in the brain.

### Developmental expression of SUMO-modified substrates in the fractionated rat brain

**Figure 2:**
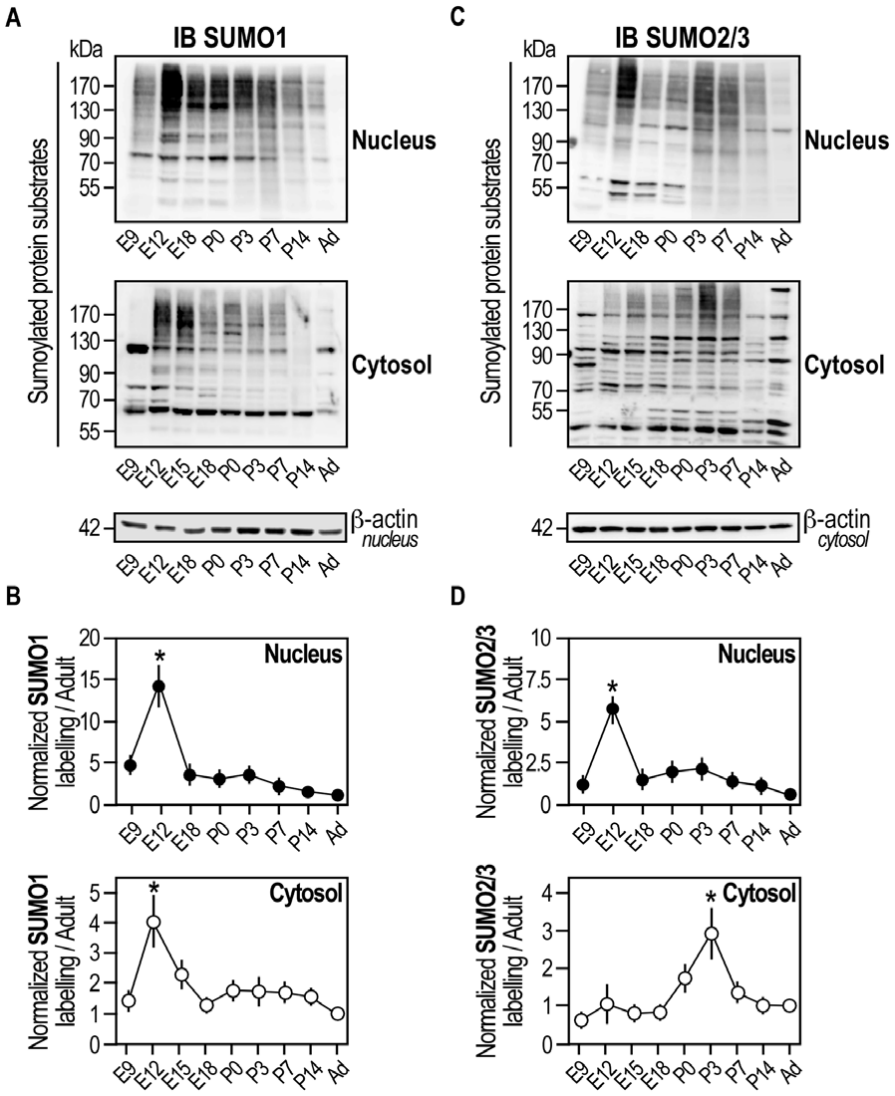
SUMO-modified substrates are developmentally regulated in the fractionated rat brain. Representative immunoblots of SUMO1- (**A**), SUMO2/3- (**C**) modified proteins in nuclear and cytosolic fractions obtained from fractionated rat brains at different developmental stages. (**B,D**) Densitometric analysis was performed using Bio1D software *(see Methods for details)*. Graphic representations normalized using ß-actin loading controls show means ± s.e.m. of at least five separate experiments. Statistical analyses were conducted with GraphPad Prism 4. One-way ANOVA was performed with a Newman-Keuls post-test for multiple comparison data sets. *p<0.001 compared with other age points.

To get further insight into the regulation of the SUMO system in the CNS, we performed rat brain fractionation experiments (**Fig. S2**) at various developmental stages to isolate nuclear, cytosolic (**Figs. 2** **,** **3**) and synaptosomal fractions (**Fig. 4**). Subcellular fractionation experiments were performed in the presence of NEM to protect synaptic proteins from desumoylation. The analysis of SUMO1- (**Fig. 2A,B**) and SUMO2/3- (**Fig. 2C,D**) conjugated protein profiles reveal that although SUMO-mediated regulation was so far mainly studied in the nucleus, the cytosolic fraction contains a very substantial proportion of sumoylated proteins. In addition, both nuclear and cytosolic sumoylated protein fractions were developmentally regulated. SUMO1-sumoylated protein expression level in nuclear and cytosolic fractions was similarly regulated with a 14.23±2.51 and 4.0±0.91 fold increase at E12 respectively, followed by a sharp decrease of sumoylation at E18 to then steadily decline with comparatively little SUMO1-modified substrates detected in adult brain (**Fig. 2B**). SUMO2/3-modified substrate profile in the nucleus was similar to SUMO1-sumoylated proteins with the highest level of expression detected at E12 (**Fig. 2D**; 5.97±0.47 fold for SUMO2/3at E12 compared to Adult). Interestingly, there was a differential regulation of SUMO2/3-sumoylated protein pattern in the cytosol with no sumoylation increase before birth but with a significant 2.89±0.90 fold increase at P3 compared to Adult. This disparity between SUMO1- and SUMO2/3-sumoylation profiles reinforced the idea that there is a SUMO-paralog specificity towards subcellular target proteins during brain development.

**Figure 3:**
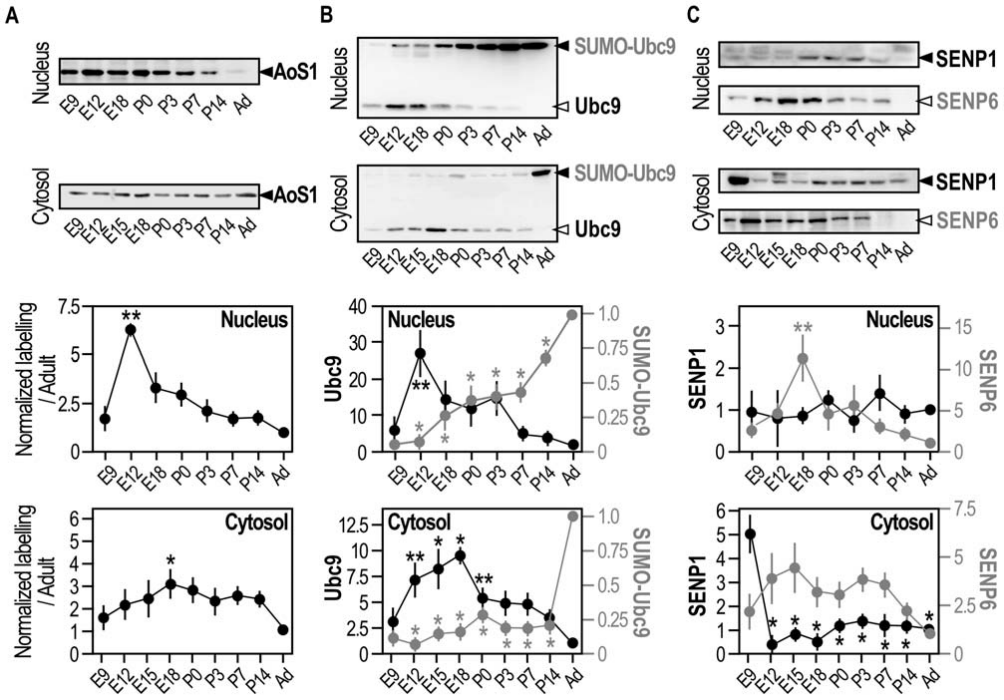
Developmental regulation of the sumoylation machinery in the fractionated rat brain. Representative immunoblots of SUMO enzymes AoS1 (**A**), Ubc9 (**B**) and SENP1/6 (**C**) in nuclear and cytosolic fractions obtained from fractionated rat brains at different developmental stages. Densitometric and statistical analyses were performed as described in figure 2 legend and graphic representations show means ± s.e.m. of five independent experiments. (**A**) **p<0.001 compared with other age points and *p<0.05 compared with adult. (**B**) **p<0.01 compared with adult and *p<0.001 compared with adult. (**C**) **p<0.01 compared with adult and *p<0.001 compared with other age points.

**Figure 4:**
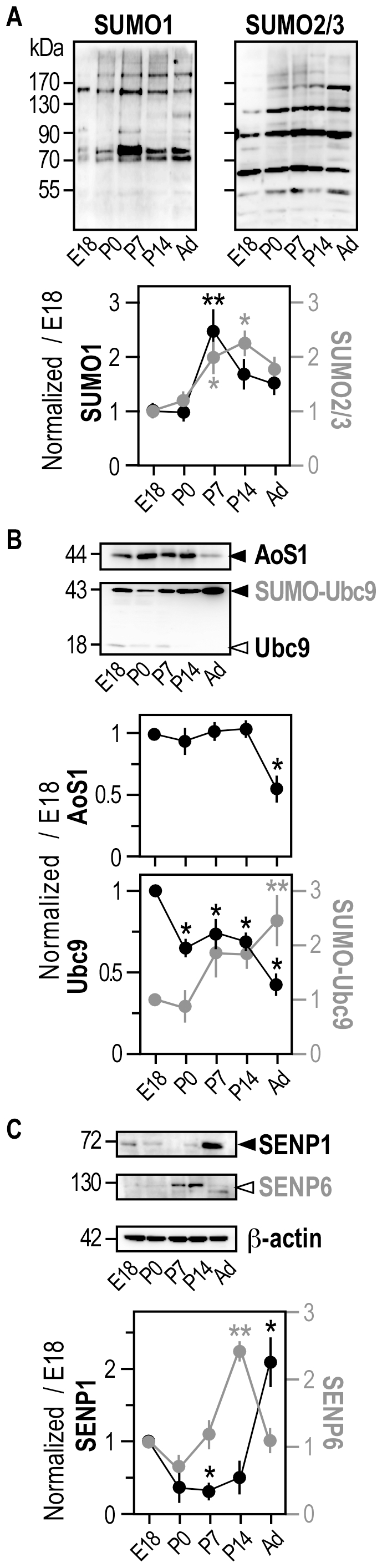
Developmental regulation of the sumoylation pathway in synaptosomal fractions. Representative immunoblots of sumoylation AoS1 (**A**), Ubc9 (**B**) and desumoylation SENP1 and SENP6 (**C**) enzymes in synaptosomal fractions obtained from fractionated rat brains at different developmental stages. Data show means ± s.e.m. of five separate experiments. One-way ANOVA was performed with a Newman-Keuls post-test for multiple comparison data sets. (**A**) **p<0.01 compared with E18 and P0 and p<0.05 compared with adult. *p<0.05 compared with P7 and P14. (**B**) **p<0.05 compared with E18 and P0 and *p<0.01 compared with other age points. (**C**) **p<0.001 and *p<0.05 compared with other age points.

### Developmental regulation of the sumoylation machinery in the fractionated rat brain

We next investigated whether the expression of sumoylation (**Fig. 3A**) and desumoylation enzymes (**Fig. 3B**) was developmentally regulated in the nuclear and cytosolic fractions described above. Our data indicate that enzymes levels are indeed differentially regulated depending on their subcellular localisation. In particular, immunodetection of the conjugation enzyme AoS1 in the nucleus was maximal at E12 with a 6.23±0.09 fold increase compared to adult nuclei. Then, the expression level of AoS1 was slowly decreased throughout the development with very little enzyme detected in adult brains. AoS1 was steadily expressed in the cytosolic fraction at all time point investigated (**Fig. 3A**). Strikingly, the two forms of the conjugating enzyme Ubc9 (*free and sumoylated SUMO-Ubc9*) showed an inverted profile. The free non-sumoylated Ubc9 was expressed early in the development, both in the nuclear and cytosolic fractions whereas the non-sumoylated Ubc9 expression levels were highest between E12 and E18 before decreasing towards the adult stage. SUMO-Ubc9 showed the converse profile with a progressive increased level of expression that reached a maximum in adults in both compartments (**Fig. 3B**).

Nuclear SENP1 was evenly expressed throughout the development with relatively low expression levels compared to the cytosolic fractions (**Fig. 3C**). The highest level of cytosolic SENP1 expression was detected at E9, the earliest time point assessed, with a significant 4.96±0.84 fold increase compared to Adult. SENP1 expression then dramatically decreased and declined steadily with comparatively little enzyme detected in adult brains. Nuclear SENP6 levels were low early in the development at E9 and showed a peak of expression at E18 with a significant 11.11±2.82 fold increase compared to adult brains. Cytosolic SENP6 expression levels were relatively steady throughout the development (**Fig. 3C**).

Our data indicate that the components of the sumoylation machinery are all expressed in synaptosomes suggesting that sumoylation might directly regulate the function of many synaptic proteins (**Fig. 4**). Moreover, synaptosomal expression levels of SUMO-modified substrates and sumoylation machinery are developmentally regulated (**Fig. 4A**). Interestingly, while the nuclear and cytosolic levels of sumoylated substrates are decreased in the adult brain, it was increased in the synaptosomal fraction (**Fig. 4**). In synaptosomes, SUMO-modified protein levels were significantly increased between E18 and P14 with a 2.46±0.45 and a 1.76±0.36 fold increase at P7 for SUMO1- and SUMO2/3-sumoylated substrates respectively (**Fig. 4A**). AoS1 expression level in synaptosomes was steady between E18 and P14 and then significantly decreased in adults (**Fig. 4B**). Ubc9 and SUMO-Ubc9 profiles were also inverted in synaptosomes with almost no detectable free Ubc9 in adult synaptosomal fractions while SUMO-Ubc9 levels increased by a significant 2.43±0.49 fold in matured brains (**Fig. 4B**). Desumoylation enzymes were also detectable in synaptosomes although there were relatively little SENPs expressed (**Fig. 4C**). SENP1 levels were low throughout the development and maximum in adult whereas SENP6 levels were higher at P7 and P14. SENP6 levels were decreased in adults with relatively more SENP1 in adult synaptosomal fractions (**Fig. 4C**).

Altogether, our data indicate that the SUMO system is highly active early in the development, predominantly in the nuclear and cytosolic compartments (**Figs. 2** **,** **3**) and that the sumoylation machinery is then redistributed in synaptosomal fractions in more matured brains (**Fig. 4**). Interestingly, *in situ* hybridization analysis revealed high expression levels of Ubc9 mRNA in various regions of the embryonic rat brain and a restricted expression of Ubc9 mRNA in adult brain mainly in cortical and hippocampal areas [30]. Sumoylation may therefore be a way to regulate protein functions important for synaptogenesis in early developmental stages and could also directly modulate synaptic transmission and/or plasticity in more mature brains.

### Synaptic redistribution of the sumoylation machinery during neuronal maturation

**Figure 5:**
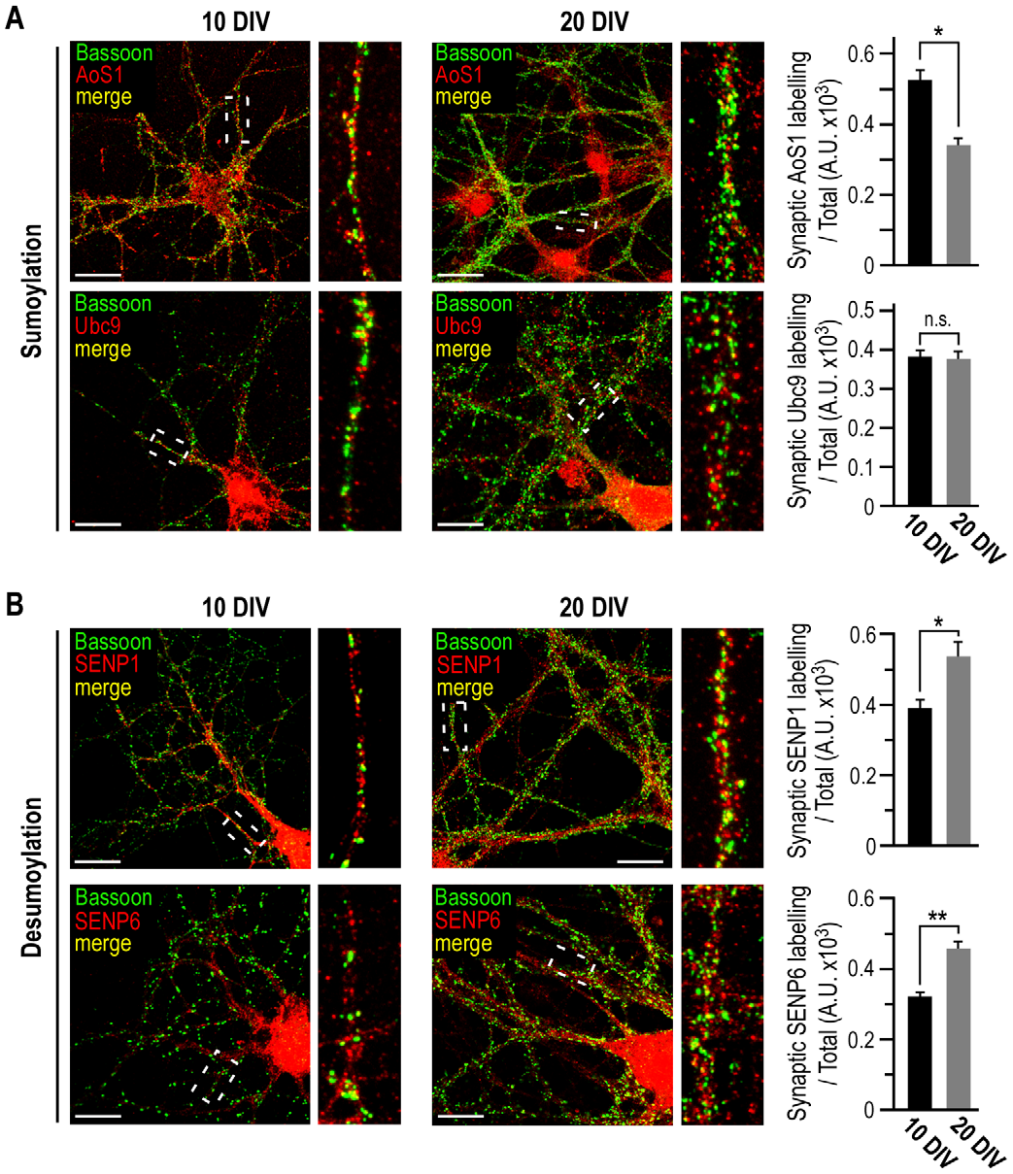
Presynaptic redistribution of the sumoylation machinery during neuronal maturation. Confocal images show the colocalisation (yellow) between the presynaptic marker Bassoon in green and in red, the sumoylation enzymes AoS1 and Ubc9 (**A**) or the desumoylases SENP1 and SENP6 (**B**) in 10 and 20 DIV cultured rat hippocampal neurons. Enlargement of hatched areas are also depicted. Scale bars, 20 µm. Quantification of presynaptic colocalisation was performed using the ImageJ software as described in the methods. Histograms represent the relative presynaptic intensity of the sumoylation machinery and each value is the mean ± s.e.m. measured from 40 cells in four independent experiments. (**A**) Student's *t*-tests, *p<0.0001; n.s., not significant. (**B**) Student's *t*-tests, *p<0.05 and **p<0.001.

To go further into the understanding of the neuronal sumoylation system, we first showed that both SUMO1 and SUMO2/3 immunoreactivities were detected in 10 and 20 DIV hippocampal neurons with intense SUMO labelling in the nucleus in agreement with the role of sumoylation in nuclear homeostasis. Punctuate SUMO labelling was also clearly detectable along the dendritic tree of both immature and mature neurons (**Fig. S3**). We next analysed the synaptic redistribution of sumoylation and desumoylation enzymes during neuronal maturation. Immunocytochemical imaging of fixed permeabilized immature 10 DIV and mature 20 DIV cultured hippocampal neurons revealed that all SUMO enzymes investigated were expressed in the nucleus, soma, dendrites and in synaptic structures (**Figs. 5** **,** **6**).

**Figure 6:**
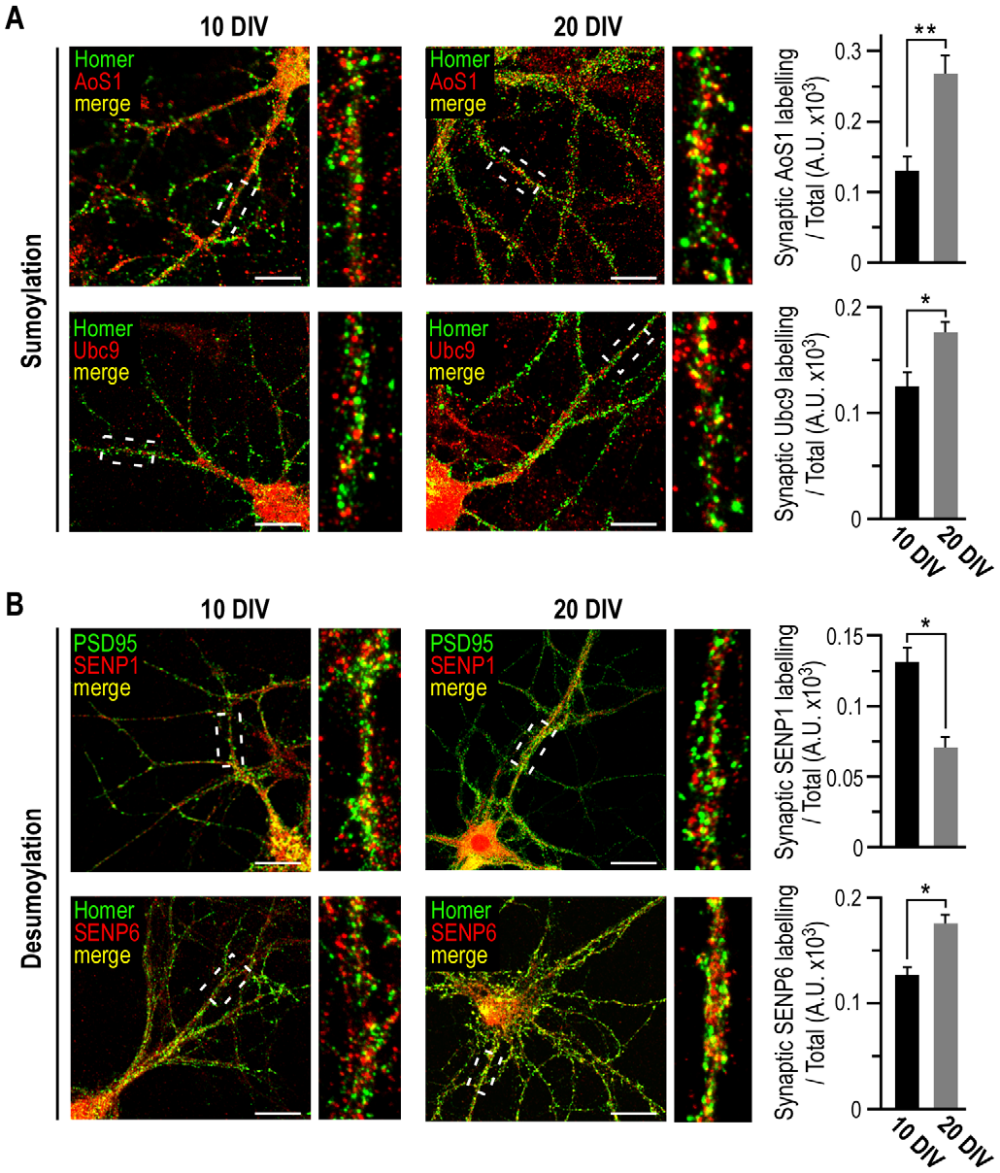
Postsynaptic relocalization of the sumoylation machinery during neuronal maturation. Confocal images show the colocalisation (yellow) between the postsynaptic markers Homer1 or PSD-95 in green and in red, the sumoylation enzymes AoS1 and Ubc9 (**A**) or the desumoylases SENP1 and SENP6 (**B**) in 10 and 20 DIV cultured rat hippocampal neurons. Enlargement of hatched areas are also depicted. Scale bars, 20 µm. Quantification of postsynaptic colocalisation was achieved using ImageJ. Histograms represent the relative postsynaptic intensity of sumoylation enzymes and each value is the mean ± s.e.m. measured from 40 cells in four independent experiments. (**A**) Student's *t*-tests, *p<0.05 and **p<0.001. (**B**) Student's *t*-tests, *p<0.01.

We measured a significant decrease of the SUMO activating enzyme AoS1 immunoreactivity in Bassoon-positive presynaptic structure (**Fig. 5A**; 0.52±0.03 at 10 DIV compared to 0.35±0.02 at 20 DIV) whereas the presynaptic distribution of Ubc9 remained unaffected by the maturation process. The desumoylating enzymes were similarly redistributed into presynaptic compartments between 10 and 20 DIV with a 1.36 and 1.44 fold increase for SENP1 and SENP6 immunoreactivity respectively (**Fig. 5B**).

We then observed that AoS1 was accumulated at Homer1-positive postsynaptic sites with a ∼2.1 fold increase (**Fig. 6A**; 0.13±0.02 at 10 DIV compared to 0.27±0.03 at 20 DIV). The conjugation enzyme Ubc9 was also targeted into dendritic spines in matured neurons with a significant 1.4 fold increase between 10 and 20 DIV (**Fig. 6A**; 0.13±0.01 at 10 DIV compared to 0.18±0.01 at 20 DIV). Interestingly, SENP1 and SENP6 show opposite redistribution profiles. While the desumoylation enzyme SENP1 localisation was decreased in dendritic spines of fully matured neurons (**Fig. 6B**; 0.13±0.01 at 10 DIV compared to 0.07±0.01 at 20 DIV), SENP6 was significantly accumulated in spines at 20 DIV (**Fig. 6B**; 0.13±0.01 at 10 DIV compared to 0.17±0.01 at 20 DIV). Our data reveal a differential redistribution of SENP enzymes in post-synaptic areas that could point out distinct target specificities for the two SENP enzymes during the maturation process.

## Discussion

Sumoylation is now seen as an important protein modification for the regulation of many proteins in the CNS. Here, we focused on two aspects of the sumoylation process in the CNS: the developmental regulation of SUMO-modified protein and sumoylation enzyme expression and the dendritic reorganization of sumoylation and desumoylation enzymes during neuronal maturation. We showed that the expression of the sumoylation machinery is developmentally regulated in the rat brain. Consistent with the known functions of sumoylation in the regulation of nuclear homeostasis, we measured high SUMO1-sumoylated substrate levels in the nucleus early in the development. Many signalling pathways have been identified recently linking neuronal activity to activity-regulated transcription factors in neurons. This regulation at the gene expression level by neuronal activity are involved in various aspects of brain development, including but not restricted to dendritic branching, synapse formation and stabilization or synapse elimination [31]. As an example, sumoylation of transcription factor Nr2e3 in developing photoreceptors was shown to promote rod photoreceptor differentiation by converting Nr2e3 into a potent repressor of cone-specific gene expression [18]. A further example comes from the elegant work from Shalizi and colleagues demonstrating the sumoylation-dependent repression of the transcription factor MEF2 (Myocyte Enhancer Factor 2) in the developing cerebellar cortex. Following neuronal activation, there is a molecular switch from MEF2A sumoylation to its acetylation leading to MEF2A activation and inhibition of synapse formation [16], [17].

For many years, sumoylation was believed to act only in the nucleus. However it is now clear that it also serves important roles outside of the nucleus (see, [3], [8], [15] for recent reviews). Here, our data are in line with these reports with highest sumoylation levels and SUMO enzyme expression early in the development followed by a change in the subcellular distribution of these enzymes with an enrichment in dendritic spines at more matured stages of the brain development. Our current results are in agreement with our previous work on the impact of sumoylation on kainate-receptor mediated synaptic transmission where we reported that the agonist-evoked sumoylation of the kainate receptor subunit GluR6 triggers their endocytosis and regulates synaptic transmission in hippocampal slices [19]#.

SENP1 and SENP6 expression levels are also developmentally regulated in the rat brain (**Fig. 3** **,** **4**) and these two desumoylases present an inversed distribution in matured neurons (**Fig. 5** **,** **6**). Interestingly, SENP1 exerts a preference towards SUMO1-sumoylated proteins while SENP6 preferentially acts on SUMO2/3-conjugated substrates [9], [10]. Despite these recent advances on the paralog specificity of the desumoylation enzymes, little is still known about the dynamic regulation of the sumoylation machinery, especially in neurons. However, the tight balance between protein sumoylation and desumoylation as well as the spatiotemporal regulation of the SUMO machinery emerge as an efficient way to dynamically modulate protein function at synapses.

Persistence of a substantial pool of synaptic SUMO1- and SUMO2/3-modified proteins and the synaptic distribution of the whole sumoylation machinery in adult brains further suggests a role for sumoylation in the regulation of synaptic function. For instance, it has been demonstrated that sumoylation of presynaptic proteins modulates neurotransmitter release. Increasing protein sumoylation by entrapping recombinant SUMO1 in synaptosomes decreased glutamate release evoked by KCl whereas decreasing sumoylation with the catalytic domain of SENP1 enhanced KCl-evoked release [29].

An additional exciting aspect in the SUMO field comes from the existing crosstalk between sumoylation and ubiquitination pathways [32], [33]. Indeed, sumoylation has been shown to compete with ubiquitination for the modification of the same target lysine residues to protect proteins from degradation. However it is now clear that this view of the interplay between these two post-translational modifications is highly reductive [33], [34], [35]. Indeed, several reports of the crosstalk between these two PTMs for the functional regulation of the same target protein are now available. As an example, several target proteins are modified with poly-SUMO chains, thereby leading to the detection by SUMO-targeted ubiquitin ligases (STUbLs), causing the proteasomal degradation of these target proteins [36], [37], [38].

In view of our data on the developmental regulation of the SUMO system in the CNS and the synaptic redistribution of the sumoylation machinery during neuronal maturation as well as the wide diversity of cellular functions regulated by this post-translational process, it is not surprising to see more and more reports implicating the sumoylation pathway in neurological disorders. A better understanding of the SUMO system in the CNS could undoubtedly help to unravel the pathogenic mechanisms of these diseases. Further work will now be required to examine these possibilities.

## Materials and Methods

### Subcellular fractionation and synaptosomal preparation

Brain fractionations (**Fig. S2**) were performed as previously described [19]#. Briefly, freshly dissected brains from *Embryonic* E9, E12, E15, E18 and *Post-natal* P0, P3, P7, P14 or adult Wistar rats (Janvier, Saint Berthevin, France) were homogenized in ice-cold sucrose buffer (10 mM Tris-HCl pH 7.4, 0.32 M sucrose, standard mammalian protease inhibitors (Sigma-Aldrich, Saint Quentin Fallavier, France) containing 20 mM NEM (Sigma-Aldrich) to protect modified proteins from desumoylation). Nuclear fractions were pelleted by centrifugation at 1,000*g* for 10 min. Post-nuclear S1 fractions were further centrifuged at 10,000*g* for 20 min to give the crude synaptosomal P2 fractions and the supernatant S2 fractions. Synaptosomes were then purified from the P2 fraction by centrifugation at 40,000*g* for 2 hours on discontinuous step gradients consisting of 1.2, 0.8, 0.32M sucrose. The synaptosomal fraction from the 0.8–1.2 M sucrose interface was collected and resuspended in lysis buffer (10 mM Tris-HCl pH 7.5, 10 mM EDTA, 150 mM NaCl, 1% Triton X100, 0.1% SDS) in presence of protease inhibitors and NEM as before. Protein concentration was determined (Bio-Rad, Marne-la-Coquette, France) and then proteins adjusted to 1 µg/µL in reducing sample buffer and boiled for 10 min.

Sample proteins (30 µg) were resolved by SDS–PAGE, electrotransferred onto nitrocellulose membranes as described before [19] and immunoblotted with the following primary antibodies: rabbit anti-SUMO1 (1/1000; [39], [40], rabbit anti-SUMO2/3 (1/240; Invitrogen, Carlsbad, CA, USA), mouse anti-Ubc9 (1/200, BD Bioscience, Rungis, France), goat anti-AoS1, rabbit anti-SENP1 and goat anti-SENP6 (1/200, Santa Cruz Biotechnology, Santa Cruz, CA, USA). Standard ß-actin controls were included in each experiment using a mouse anti-ß-actin antibody (Sigma, Saint Quentin Fallavier, France). Intensities of bands were quantified using Bio1D software (Vilber-Lourmat, Marne-la-Vallée, France). Densitometric values for SUMO-modified proteins (ranging from 35 and 250 kDa) measured from each entire lane were compared to the corresponding adult's brain fractions (figures 2 and 3) or to the corresponding E18 values (figure 4).

### Dispersed hippocampal neuronal cultures

Primary hippocampal cultures were prepared from E18 pregnant Wistar rats as previously described [41], [42]. Cells were plated in Neurobasal medium (Invitrogen, Villebon sur Yvette, France) supplemented with 2% B27 (Invitrogen), 0.5 mM glutamine, 12.5 µM glutamate and penicillin/streptomycin on 24-mm glass coverslips pre-coated with poly-L-lysine (0.1 mg/mL). Neurons (100,000 cells per coverslip) were then fed once a week for 3 weeks in Neurobasal medium (Invitrogen) supplemented with 2% B27 (Invitrogen) and penicillin/streptomycin.

### Immunocytochemistry

Hippocampal neurons were rinsed twice in phosphate-buffered saline (PBS) and then fixed with PFA 4% in PBS for 10 min at room temperature (RT). Fixed cells were permeabilised for 20 min in PBS containing 0.1% Triton X100 and 10% Horse Serum (HS) at RT. Neurons were then incubated as indicated on the figures with a combination of goat anti-AoS1, mouse anti-Ubc9 or goat anti-SENP6 antibodies (1/50), rabbit anti-Homer1 (1/200; Synaptic System, Gottingen, Germany), rabbit anti-Bassoon (1/200; Stressgen), rabbit anti-SENP1 (1/50) and mouse anti-PSD-95 (1/100; NeuroMab, Davis, CA, USA) overnight at 4°C in PBS containing 0.05% Triton X100 and 5% HS. Cells were washed three times 10 min in PBS and incubated with the appropriate secondary antibodies conjugated to either Alexa488 or Alexa594 in PBS containing 5% HS with 0.05% Triton X100 for 1 h at RT, washed three times in PBS and mounted with Mowiol (Sigma) before confocal examination.

### Image analysis

Sequential confocal images (1024×1024 pixels) were acquired with a 63× oil-immersion lens (Numerical Aperture, 1.4) on an inverted TCS-SP5 confocal microscope (Leica Microsystems, Nanterre, France). Z-series of 7–8 images of randomly chosen dendrites were compressed into two dimensions using the maximum projection algorithm of the Leica software. Quantification was performed using the ImageJ 1.42 software (NIH, USA) and the synaptic enzymatic staining was measured with the use of an in-house ImageJ macro. Briefly, confocal images of synaptic marker were used to produce masks after an automated intensity threshold. Masks were applied to the corresponding sumoylation/desumoylation enzyme images and the fluorescence intensity within the synaptic area was measured.

### Statistical analysis

The N used for statistical analysis was either the number of animals, the number of experiments or the number of cells and is indicated in the figure legends. Statistical analyses were calculated using Prism 4 (GraphPad software, Inc., La Jolla, CA, USA). Data were expressed as mean ± s.e.m.. Unpaired Student's *t*-tests or one-way ANOVA were performed with a Newman-Keuls post-test for multiple comparison data sets when required.

## Supporting Information

Figure S1 **Ubc9 is highly sumoylated in adult rat brain homogenates.** (**A**) Immunoprecipitation experiments using anti-Ubc9 antibody revealed that Ubc9 is abundantly sumoylated in adult brain homogenates. Control IgG antibodies were also used here as a control for immunoprecipitation. Heavy (hc) and light (lc) IgG chains are indicated on the figure. (**B**) Ubc9 immunoblot on brain protein extracts obtained in the absence or in the presence of 20 mM NEM (*to protect SUMO-modified proteins from desumoylation during cell lysis*) showed that the amount of sumoylated Ubc9 is reduced in the absence of NEM with the concurrent increase of the 18 kDa non-sumoylated Ubc9 band intensity further demonstrating that Ubc9 is sumoylated in neurons.
(TIF)

Figure S2 **Subcellular rat brain fractionation.** (**A**) Schematic of the subcellular fractionation protocol utilized to collect the nuclear, cytosolic and synaptic fractions. (**B**) Immunoblots showing the synaptic PSD-95 protein, nuclear HDAC3 marker and control ß-actin labelling to assess brain fractionation.
(TIF)

Figure S3 **SUMO1 and SUMO2/3 labelling in immature and mature rat hippocampal neurons.** SUMO1 and SUMO2/3 labelling in 10 or 20 DIV rat hippocampal neurons. Note that the SUMO labelling is intense within the nucleus in agreement with the role of sumoylation in the control of nuclear homeostasis. Interestingly, SUMO immunoreactivity was also detected as a punctuate staining in the dendritic tree of immature and mature neurons. Scale bars, 20 µm.
(TIF)
