# Supplementary figures and images for: Developmental Regulation and Spatiotemporal Redistribution of the Sumoylation Machinery in the Rat Central Nervous System
Source: PLoS One. 2012 Mar 16;7(3):e33757. doi: 10.1371/journal.pone.0033757 (PMC3306303; doi:10.1371/journal.pone.0033757)

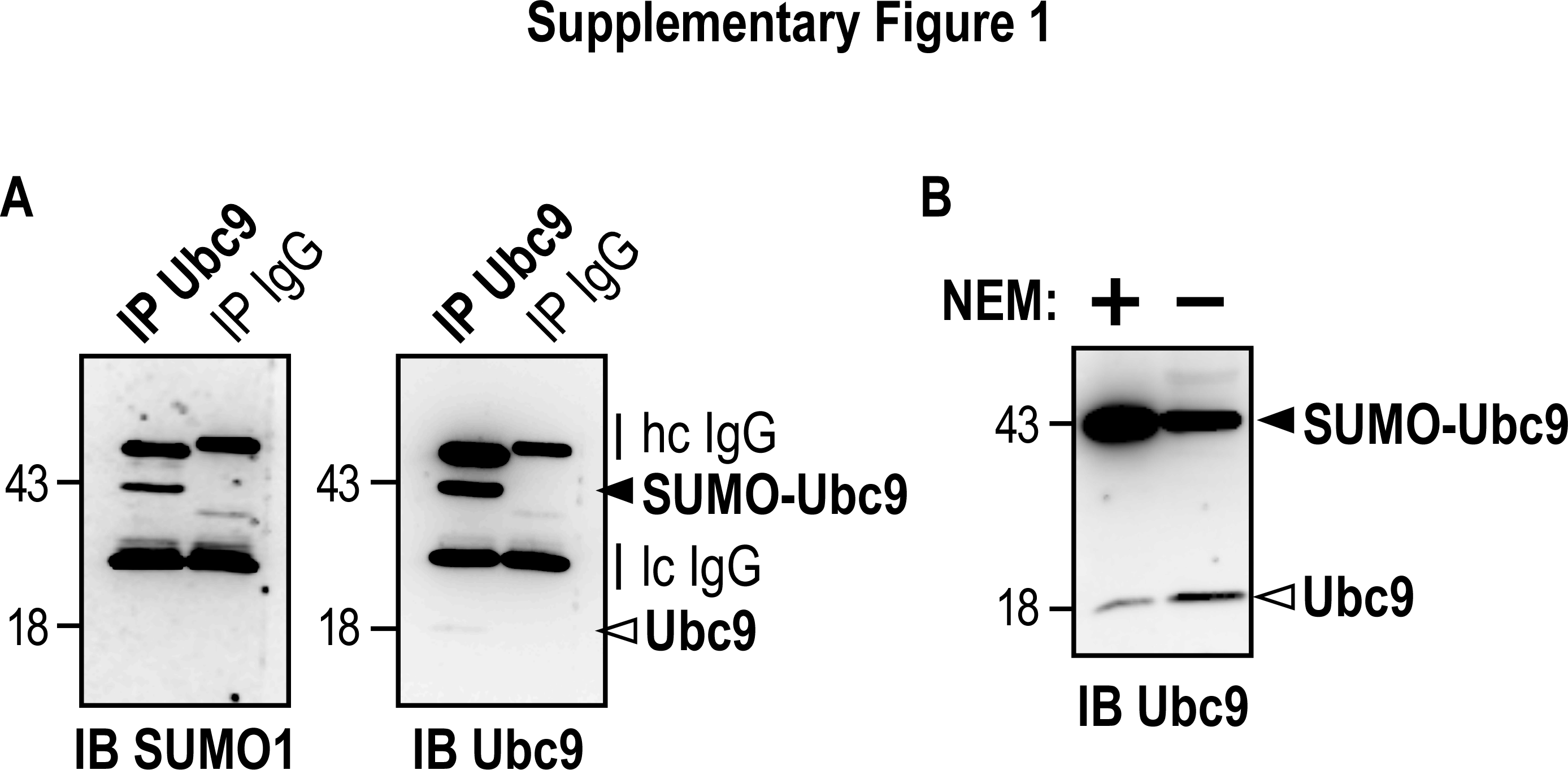

Supplement: Figure S1 — Ubc9 is highly sumoylated in adult rat brain homogenates. (A) Immunoprecipitation experiments using anti-Ubc9 antibody revealed that Ubc9 is abundantly sumoylated in adult brain homogenates. Control IgG antibodies were also used here as a control for immunoprecipitation. Heavy (hc) and light (lc) IgG chains are indicated on the figure. (B) Ubc9 immunoblot on brain protein extracts obtained in the absence or in the presence of 20 mM NEM (to protect SUMO-modified proteins from desumoylation during cell lysis) showed that the amount of sumoylated Ubc9 is reduced in the absence of NEM with the concurrent increase of the 18 kDa non-sumoylated Ubc9 band intensity further demonstrating that Ubc9 is sumoylated in neurons. (TIF) [file pone.0033757.s001.tif]

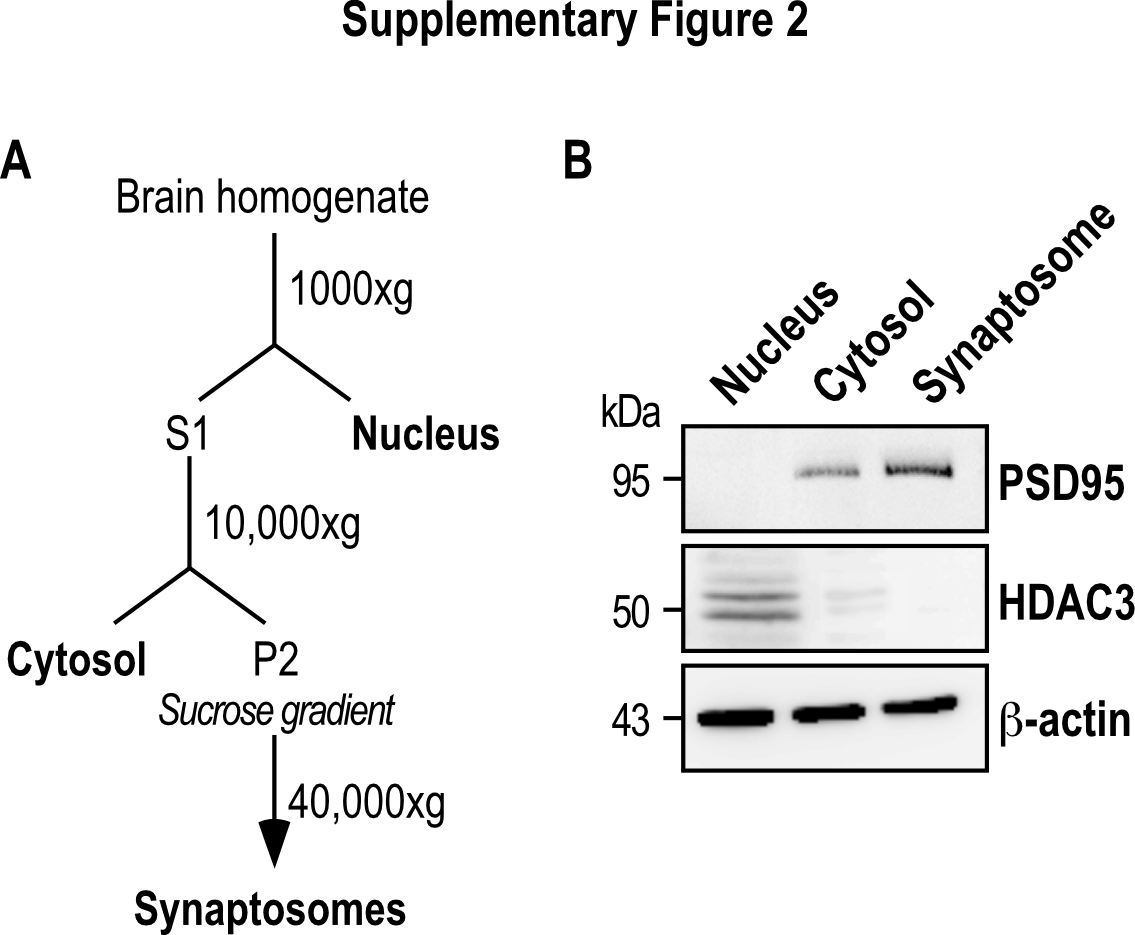

Supplement: Figure S2 — Subcellular rat brain fractionation. (A) Schematic of the subcellular fractionation protocol utilized to collect the nuclear, cytosolic and synaptic fractions. (B) Immunoblots showing the synaptic PSD-95 protein, nuclear HDAC3 marker and control ß-actin labelling to assess brain fractionation. (TIF) [file pone.0033757.s002.tif]

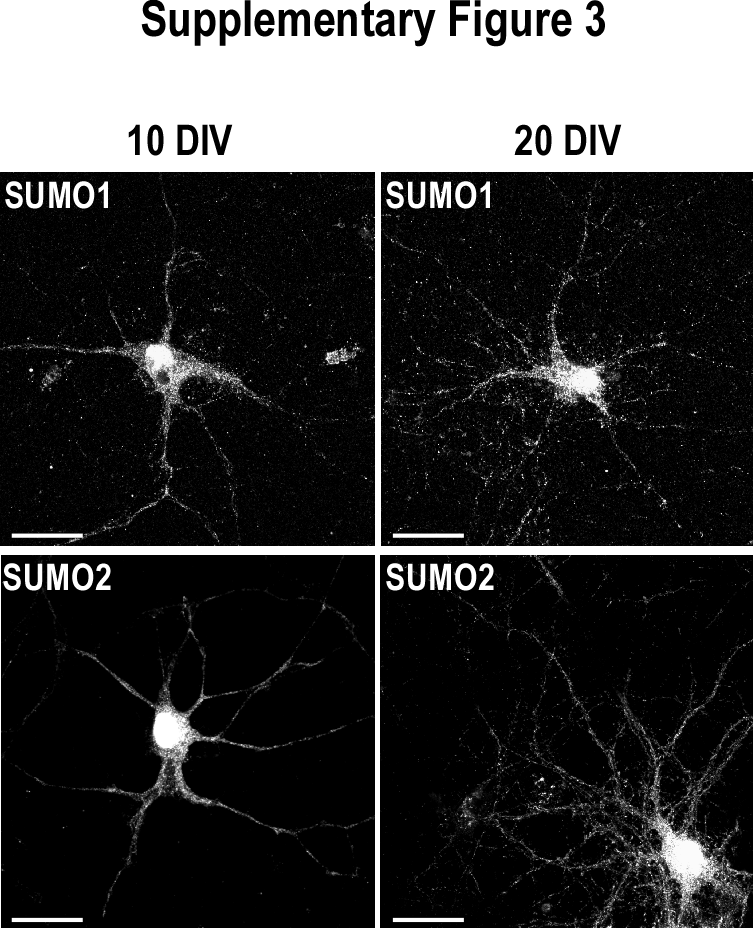

Supplement: Figure S3 — SUMO1 and SUMO2/3 labelling in immature and mature rat hippocampal neurons. SUMO1 and SUMO2/3 labelling in 10 or 20 DIV rat hippocampal neurons. Note that the SUMO labelling is intense within the nucleus in agreement with the role of sumoylation in the control of nuclear homeostasis. Interestingly, SUMO immunoreactivity was also detected as a punctuate staining in the dendritic tree of immature and mature neurons. Scale bars, 20 µm. (TIF) [file pone.0033757.s003.tif]
